# Mechanical Behaviour of ABS-Fused Filament Fabrication Compounds under Impact Tensile Loadings

**DOI:** 10.3390/ma12081295

**Published:** 2019-04-19

**Authors:** Caterina Casavola, Alberto Cazzato, Vincenzo Moramarco, Gilda Renna

**Affiliations:** Dipartimento di Meccanica, Matematica e Management (DMMM)—Politecnico di Bari—Viale Japigia 182–70126 Bar, Italy; caterina.casavola@poliba.it (C.C.); alberto.cazzato@poliba.it (A.C.); gilda.renna@poliba.it (G.R.)

**Keywords:** fused filament fabrication, fused deposition modelling, tensile impact, orthotropic materials, ABS

## Abstract

In the Fused Filament Fabrication (FFF) process, the part is built as a layer-by-layer deposition of a feedstock filament material. The continuous improvements of the FFF have changed the main purpose of this technique from rapid prototyping to a rapid manufacturing method. Then, it is fundamental to determine the material properties of FFF parts as a function of the service load. The impact loads and, in particular, a high strain rates tensile impact can be a critical issue in FFF part and, in general, for plastic materials. The aim of the present work is to characterise the mechanical behaviour of FFF parts under tensile impact loads. To this purpose, three different orientations (i.e., 0°, 45° and 90°) both single- and multilayer specimens, have been printed. Finally, the influence of the impact speed on the mechanical behaviour has also been tested under three different values of speed (3.78 m/s, 3.02 m/s and 2.67 m/s). The results show that the FFF parts are influenced by the raster orientation, confirming the orthotropic behaviour also under dynamic loads, while the variation of impact speed, on peak force and absorbed energy, is limited.

## 1. Introduction

**Figure 1:**
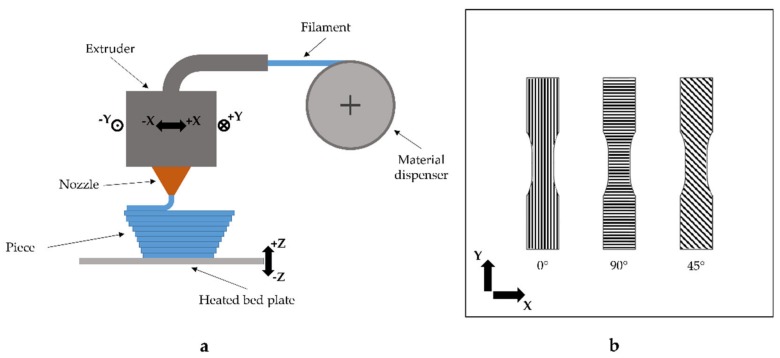
Schematic representation of the Fused Filament Fabrication (FFF) process (**a**) and build orientation (**b**).

One of the most employed 3D printing techniques, in both consumer and enterprise environments, is the Fused Filament Fabrication (FFF). This process, developed by Stratasys Inc. in the early 1990s with the commercial naming of Fused Deposition Modelling (FDM), has been employed in many fields such as aerospace, medical, construction and cultural [1,2]. Initially the main applications were the kinematic functionality testing, design verification and fabrication of models for visualisation [3]. However, at present, FFF also has a significant role in the rapid production of finished parts [3]. As in other applications of 3D printing technologies [4], in this process the part is built as a layer-by-layer deposition of a feedstock filament material. The raw material is partially melted, extruded and deposited onto the previously built model by a numerically controlled heated nozzle [1] (Figure 1a). After the deposition, the material cools and sticks with the surrounding material and the machine can move the nozzle upward, along the z-axis, for the deposition of the next layer. Initially, FFF printers have been able to build parts only in acrylonitrile–butadiene–styrene (ABS) and polylactic acid (PLA). The latter one, compared to ABS, has stronger mechanical resistance and lower coefficient of thermal expansion. The last property improves the printability of the material because it reduces the delayering problems and the warp effect during the printing phase. Consequently, the residual stresses in the material should also be reduced [5,6,7]. Nowadays, many others materials have been employed and developed, e.g., metal [8], ceramics [9], bioresorbable polymer (PCL) [10], metal/polymers mixture materials [11] and short fibre composites [12].

The principle of the FFF technology has great potential because it allows the fabrication of complex 3D parts directly from a computerised solid model. Moreover, in recent years, the continuous improvements of the FFF have allowed to change the main purpose of this technique from rapid prototyping to a rapid manufacturing method [3], building single pieces or low volume products, e.g., replacement parts for none widespread systems. However, as it has been shown by Casavola et al. [13], the final FFF part shows an orthotropic behaviour similar to a laminate orthotropic structure. Accordingly, it is of fundamental importance to determine the material properties by changing the direction of the deposition and, as a function of the service load, accurately design the deposition strategy of the printed parts. Research activity regarding the properties of FFF components has gained interest over the last years and many papers have been published about the mechanical properties of FFF parts [14,15,16,17,18,19,20]. Tymrak et al. [14] tested specimens with different deposition strategy under tensile loads. These specimens have been made by several, both open-source and professional grade, 3D printers. The results showed that 3D printed components from open-source printers were comparable in tensile strength and elastic modulus to the parts printed on commercial 3D printing systems. Moreover, specimens printed with 0.2 mm layer height had the greatest tensile strength, while specimens at 0.4 mm layer height had the greatest elastic modulus. Between the 0°/90° and +45°/−45° orientations, +45°/−45° was the strongest, while 0°/90° had the greater elastic modulus. Ziemian et al. [15] studied the mechanical properties, i.e., tensile, compression and three-point bend, of ABS specimens fabricated by FFF. The results showed an anisotropic behaviour that was significantly influenced by the orientation of the layer beads. Tension and three-point bend tests indicated that the ultimate and yield strengths were the largest for the 0° raster orientation, followed by the +45°/−45°, 45° and 90° orientations. The compression test data indicated that the 45° raster specimens were significantly weaker in compression than the other raster orientations. Ahn et al. [19] carried out several tests to study the effects of air gap, raster orientation, bead width and ABS colour on tensile and compressive strengths. They showed that the air gap and the raster orientation had an important effect on the tensile strength but the other parameters had negligible effects. Moreover, the compressive strength was not influenced in a noticeable way by the factors studied. Finally, also Lee et al. [20] concluded that raster angle, layer thickness and air gap influence on the elastic performance of flexible ABS parts.

The impact loads and, in particular, high strain rate tensile impact, can be critical issues in FFF parts and, in general, for plastic materials [21,22]. However, limited research has been done in understanding the impact mechanical behaviour of FFF 3D printed parts and, to the authors’ knowledge, no research work has been done about the tensile impact strength of FFF. Sood et al. [16], using the response surface methodology, analysed the functional relationship between the Charpy impact strength and several factors, e.g., build orientation, layer thickness, raster angle and air gap. Tsouknidas et al. [23] studied the impact absorption capacity of 3D-printed structures fabricated by FFF. Results show that the concentric filling performs better compared to the other filling typology while the rectilinear patterns and bulk filling exhibited the worse compressive response. Also, Es-Said et al. [24] examined the effect of layer orientation on the impact strength of rapid prototype ABS samples. The Izod impact test data indicated that the 0° orientation samples had the highest absorbed energy by an order of magnitude over the 90° orientation. Although the Charpy and Izod tests can provide important information about the effect of the orientation on the absorbed energy, no information about the effect of the loading speed is given. However, as is well known, the response at different deformation rates is generally a significant issue of the mechanical behaviour of plastic materials. Furthermore, tensile impact tests can be performed on specimens with a variable number of layers, providing information about the effect of the stacking.

The aim of the present work is to characterise the mechanical behaviour of FFF parts under tensile impact loads. To this purpose, the influence of the orthotropic characteristic of the material will be evaluated by designing and testing specimens with different orientations, i.e., 0°, 45° and 90° (Figure 1b). In order to fully characterise the impact behaviour of FFF parts, both single layer and multilayer specimens will be assayed. Finally, to prove the influence of impact speed on the mechanical behaviour, three different values of speed will be used.

## 2. Materials and Methods

In this work, ABS specimens obtained from a specific FFF process were tested under rapid dynamic tensile loads with relatively high initial impact velocities. In order to study the orthotropic mechanical behaviour of FFF compounds during the tensile impact tests, both single-layer and multilayer specimens were tested. The principal mechanical, thermal and physical properties of the used ABS filament, as declared by product datasheet, have been listen in Table 1.

The thickness of the single-layer sample is 0.35 mm, while the multilayer, made of 6 layers, has a thickness of 2.1 mm. Moreover, the tests were repeated for three layer orientations, i.e., 0°, 45° and 90°, where a layer with a 0° raster angle have the deposited beads parallel to the major side of the specimen. In addition to the influence of the raster orientations and the comparison between single and multilayer, the influence of the impact speed was studied. In order to carry out this part of the study, the energy of the striker has been fixed to 25 J and, employing additional masses, the striker speed has been changed. The Table 2 summarises the test parameters. In order to have the possibility to statistically analyse the results, ten samples for each typology of specimen were printed. Taking into account the three raster angles, a total of 90 specimens were made.

The shape of the specimens was designed according to the standard ISO 8256 [25], type III for the impact tensile tests. The solid model was designed using a 3D cad and it was sliced employing the open source software Slic3r (v. 1.1.7).

The samples were printed employing a RepRap Prusa i3 (Prusa Research, Prague, Czech Republic) with a Marlin firmware and a 0.4 mm nozzle. Some parameters were kept constant for every specimen and have been reported in Table 3. The bed temperature was set to 90 °C and the nozzle temperature is 225 °C. Moreover, the specimens were fabricated with the minimum dimension of the part perpendicular to the build platform.

In Table 3, the air gap is the distance between two, adjacently deposited beads of the same layer; the layer thickness and the bead width are respectively the height and the width of a deposited filament. The number of contours represents the number of edges that have been deposited before filling the inner part by inclined beads.

**Figure 2:**
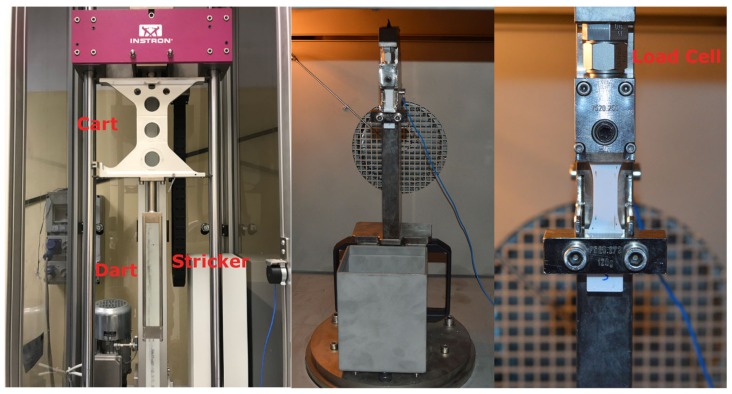
Striker bar system and an example of specimen mounted in the grips in the temperature-controlled chamber.

For the tensile impact tests of both single layer and multilayers, an Instron CEAST 9350 drop tower with the software Visual Impact for the data acquisition and manipulation was used. According to the machine setup, the A method of the ISO 8256 and a 120 g crosshead was employed. The system was equipped with a 2.2 kN piezoelectric load cell, placed just at interface with the specimen (Figure 2). Furthermore, the system is instrumented with a speed sensor that records the impact velocity. In order not to introduce local stress concentration due to excessive clamping force, the specimens were mounted on the load train (Figure 2) employing a torque of 2.5 Nm on the clamp screws.

**Figure 3:**
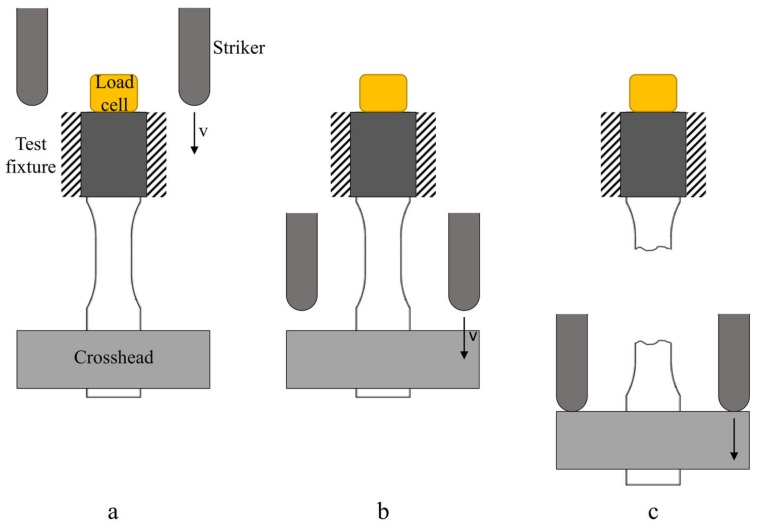
Schematic representation tensile impact test (A Method): (**a**) initial set up, (**b**) striker displacement and (**c**) break of the specimen.

In Figure 3 a schematic representation of the impact test is shown. One end of the specimen is clamped to the test fixture while at the other end a crosshead is fixed. When the striker hits, the crosshead breaks the specimen. The striker, made of steel, is composed by two different sections (Figure 2): the Y-shape dart (41 cm length) and the cart to add the mass of the striker.

Using the recorded load history *F(t)* and the impact velocity *v_i_*, it is possible calculate the absorbed energy *E_s_* (not corrected) as

(1) $$E_{S}=\int_{0}^{I_{max}}F\left(I\right)dI$$

where *I* is the displacement that can be calculate as a function of the time as

(2) $$I\left(t\right)=v_{i}t-\frac{1}{m}\int_{0}^{t}\left[\int_{0}^{t}F\left(t\right)dt\right]dt+\frac{1}{2}gt^{2}$$

where *m* is the mass of the striker and *g* is the acceleration of gravity.

This value of energy should be corrected to take into account the increase of the mass of striker after the rupture of the specimen due to the crosshead. According whit ISO 8256, the corrected absorbed energy *E_c_* is equal to

(3) $$E_{c}=E_{S}-E_{q}$$

where *E_q_* is the energy due to the kinetic energy of the crosshead that can calculated as

(4) $$E_{q}\approx \frac{3}{2}E_{max}\mu$$

where *E_max_* is the maximal impact energy and *μ* is the ratio between the mass of the crosshead and the mass of the striker (*m_cr_*/*m*).

Furthermore, quasi-static tensile tests were conducted on single and multilayer specimens according to ASTM D638-10 in order to underline the orthotropic behaviour of the material [13].

## 3. Results

**Figure 4:**
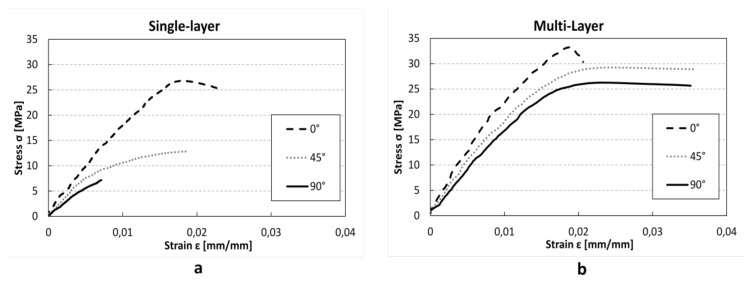
Representative quasi-static tensile tests for single (**a**) and multilayer (**b**) specimens at each raster orientation.

Initially, in order to evaluate the orthotropic behaviour of the FFF printed material, quasi-static tests were conducted both on single and multilayer specimens. Figure 4 shows the stress–strain behaviours at different raster angles. Both for single- and multilayer specimens a clear influence of print direction on linear elastic modulus was found. In fact, Young’s modulus changes from 1.79 GPa at 0° to 1.15 GPa at 90° in single layer specimens (−35%), and from 1.96 GPa at 0° to 1.55 GPa at 90° (−16%) in multilayer specimens (Table 4). However, the multilayer specimens show a general increase of stiffness in comparison with the single-layer specimens, independently from print direction (+9.5% at 0°; +27% at 45°; +42% at 90°), and a reduction of orthotropic behaviour. This behaviour could be explained with the partial overlap of the different layer and consequently the reduction of the internal voids that improve the mechanical characteristic of the material. In 45° and 90° specimen production the time between the deposition of a cordon and the subsequent cordon is lower, allowing better bonding between them.

Also, UTS shows direct correlation with raster angle (Table 4). Furthermore, similarly to linear elastic modulus, multilayer specimens show an increase of UTS in comparison with single-layer specimens. Finally, while in single-layer tests the absorbed energy shows a decrease with the increase of the angle between the load direction and the raster angle, in the multilayer test the maximum was found at 45° raster angle. The multilayer specimens with 45° orientation, in fact, show clear elasto-plastic behaviour (Figure 4b), which is probably due to the reduction of the internal voids compared with 0° specimens.

Subsequently, tensile impact tests were carried out. Using the Visual Impact software, the data from the machine, i.e., the Force F [N] and the elongation Δl [mm], was acquired. This data was processed in order to calculate the stress σ [MPa] and the strain ε [mm/mm]. The results of the tensile impact tests are presented into two separate sections. Initially the single-layer tests will be analysed and then the multilayer results will be shown

### 3.1. Single-Layer Samples

**Figure 5:**
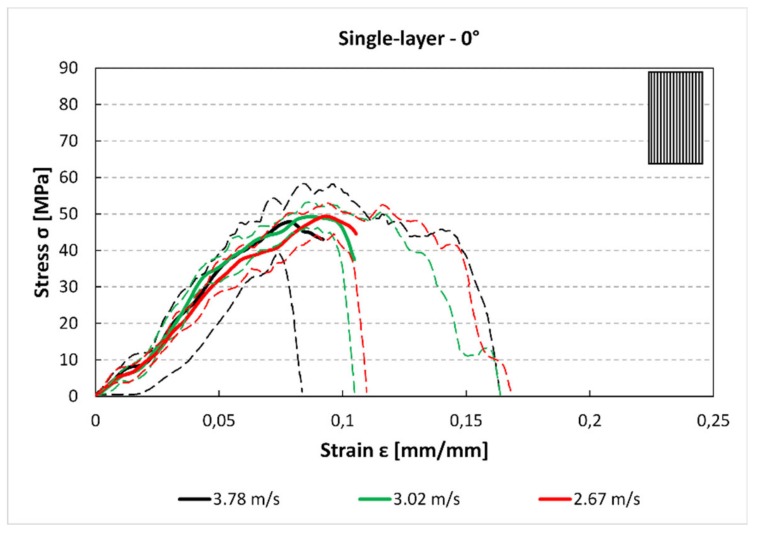
Single-layer stress–strain curve for 0° specimen and for each impact speed.

**Figure 6:**
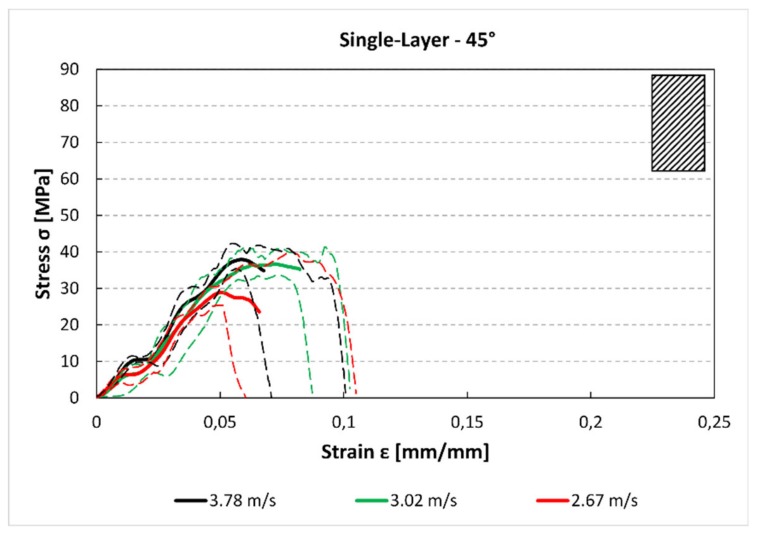
Single-layer stress–strain curve for 45° specimen and for each impact speed.

**Figure 7:**
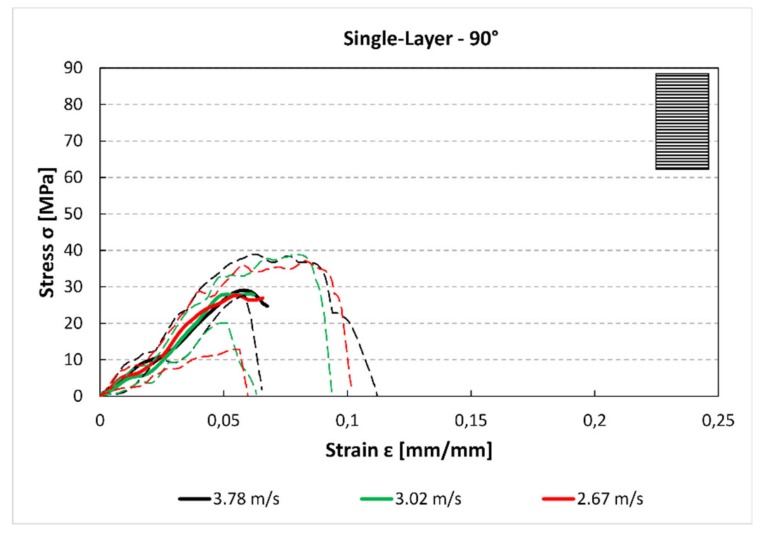
Single-layer stress–strain curve for 90° specimen and for each impact speed.

In Figure 5, Figure 6 and Figure 7 the average stress–strain curves and the experimental data corridors at different impact speeds and for each raster orientations are reported. These images, although present some limitation (e.g., the average curves can be considered only up to the maximum), allowing for some considerations about the mechanical behaviour of component obtained by FFF under high strain rate loads. In Figure 5, the stress–strain curve for the 0° specimens is plotted (engineering stress and strain). From the trends it can be highlighted that the impact speed has no influence on both the maximum stress. Indeed, the peaks of the three curves are near 50 MPa for each speed.

For the single-layer 45° specimens (Figure 6), there is a reduction of the maximum stress and strain compared to the 0° samples (Figure 5). Moreover, the changes due to the impact speed are not significant between 3.78 m/s and 3.02 m/s, while for 2.67 m/s, there is a reduction of maximum stress. This could be due to secondary flexion loads, generated by asymmetry of specimen that can reduce the maximum stress especially at lower velocity.

In Figure 6, the trends for the 90° samples are reported. There is a further reduction in maximum stress compared to the other stacking sequences. Also in this case, the behaviour of the printed material is not influenced by the impact speed because the maximum stress is comparable for the three speeds.

**Figure 8:**
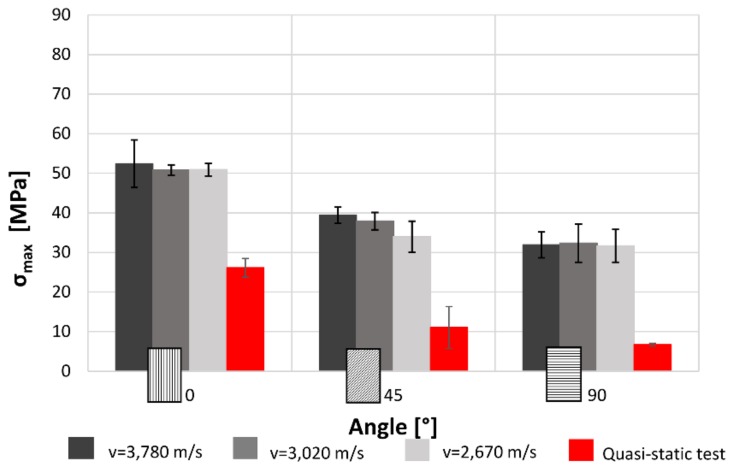
Effect on the maximum stress of the raster angles and impact speeds for single-layer specimens.

Furthermore, the tests show that increasing the raster angle there is a reduction of the maximum stress (Figure 8). However, the variation among the three speeds can be neglected because the stress values are mostly the same for each speed. In addition, the results from quasi-static tensile test are lower than the dynamic values.

While the strong correlation between the print direction and maximal stress was expected, due to the orthotropic behaviour of this kind of material, the independence from the loading speed is probably related with fracture mechanism of the specimens that is primarily fragile.

**Figure 9:**
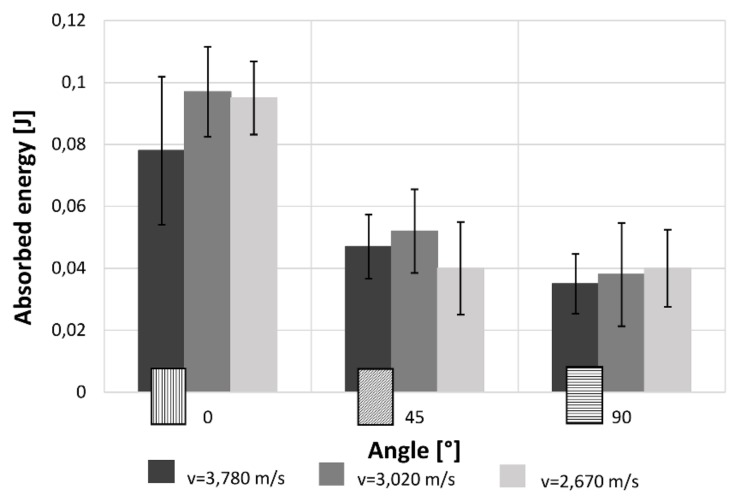
Effect on the absorbed energy of the raster angles and impact speeds for single-layer specimens.

Figure 9 highlights that the trend of the absorbed energy is comparable to the maximum stress trend. Indeed, when increasing the raster angle there is a great reduction of the absorbed energy. Also in this case, the alterations due to the impact speeds considering the uncertainty can be considered not relevant from a statistical point of view. The recorded values are lower in comparisons with that measured under quasi-static condition, except for the 90° raster angle. This could probably be due to the extremely fragile behaviour of the 90° samples even in quasi-static conditions.

**Figure 10:**
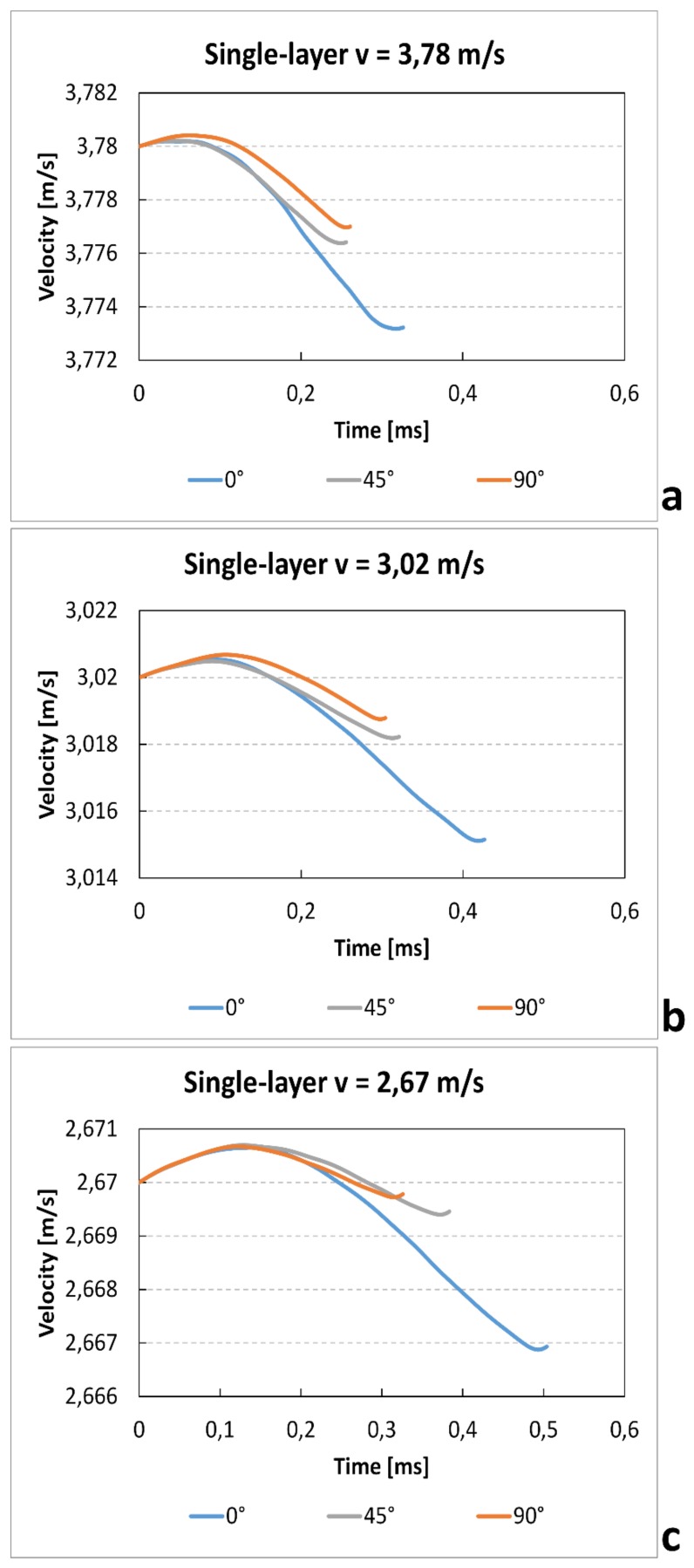
Single-layer velocity–time curves for different raster angle and velocity.

Figure 10 shows the velocity–time curves at the variation of the orientation angle and of the impact velocity. The apparent small increase of the velocity after the zero time (<<1%) is due to the manual positioning of the photocell that permit to record the initial impact velocity. The results show a clear increase of time test and a reduction gradient velocity with increase of impact speed. These results are coherent with the reduction of absorbed energy that is almost independent of speed variation. Furthermore, for all impact velocities, the specimens in the 0° direction show a longer test duration. This is probably due to the capacity of the bead to increase the breaking deformation.

**Figure 11:**
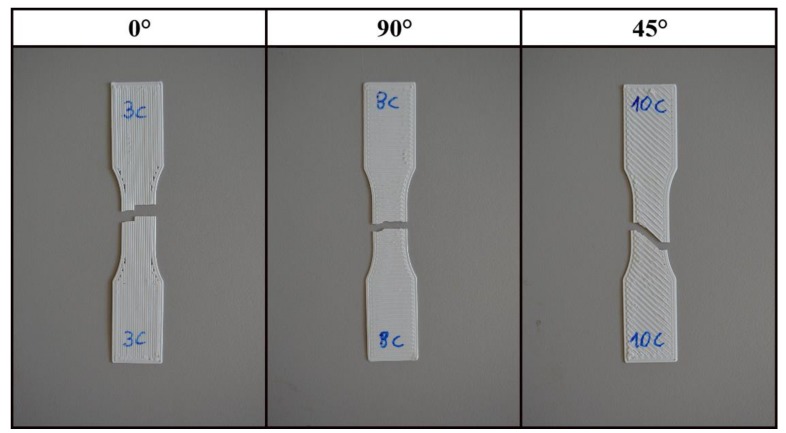
Fracture of single-layer specimens.

In Figure 11, an example of the fracture of the single-layer specimens is reported. The 0° specimens show tensile failure of individual fibres resulting in the highest maximum stress and absorbed energy among the tested raster angles. The 90° specimens had the lowest maximum stress and absorbed energy mainly because the applied loads weighs on the bonding between fibres. In a similar way for the 45° specimens, the crack propagation moves inside the weak part of the specimens that is in the bonding between two beads.

### 3.2. Multilayer Samples

**Figure 12:**
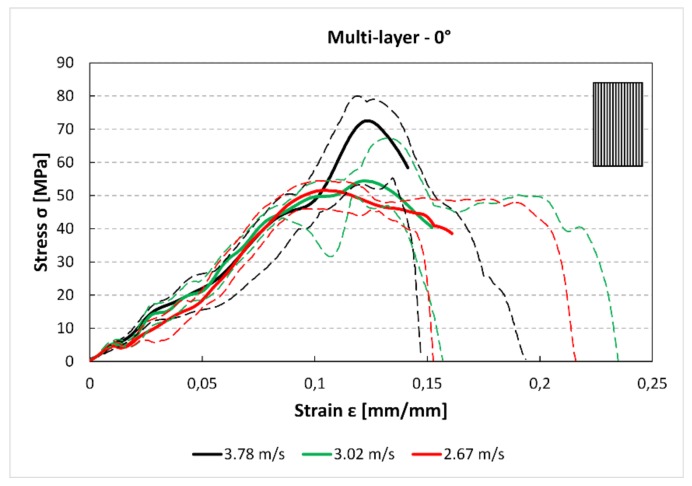
Multilayer stress–strain curves for 0° specimens and for each impact speed.

The same experimental campaign of the single-layer specimens has been carried out on the multilayer samples. In Figure 12 can be observed as a higher impact speed produces higher stress in the stress–strain curves. While this difference is significant between the 3.78 m/s and 3.02 m/s speeds, the comparison between the two lower speeds, i.e., 3.02 m/s and 2.67 m/s, does not highlight this clear difference. Indeed, the 3.78 m/s curve though shows higher stress values has smaller deformation than the other two curves.

**Figure 13:**
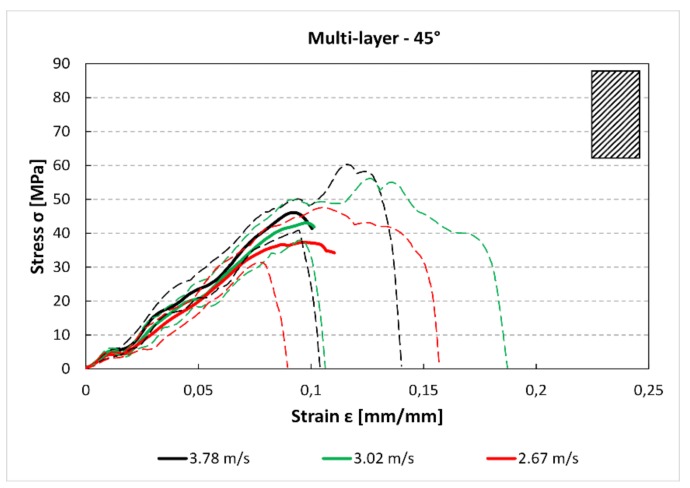
Multilayer stress–strain curves for 45° specimens and for each impact speed.

**Figure 14:**
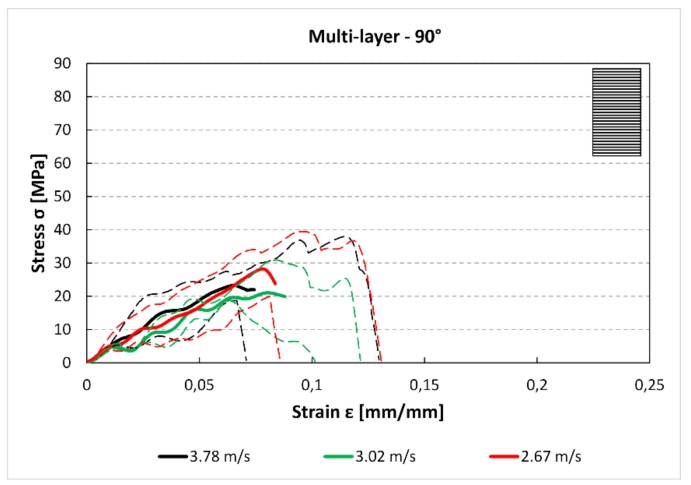
Multilayer stress–strain curves for 90° specimens and for each impact speed.

The comparison between Figure 12 and Figure 13 shows that there is a reduction of the maximum stress and the absorbed energy between the 0° and 45° specimens. Moreover, for the 45° specimens, there is a small reduction of the maximum stress between the 3.78 m/s and 2.76 m/s curves. In Figure 14, it can be observed a further reduction of the maximum stress compared to the previous 0° and 45° cases.

**Figure 15:**
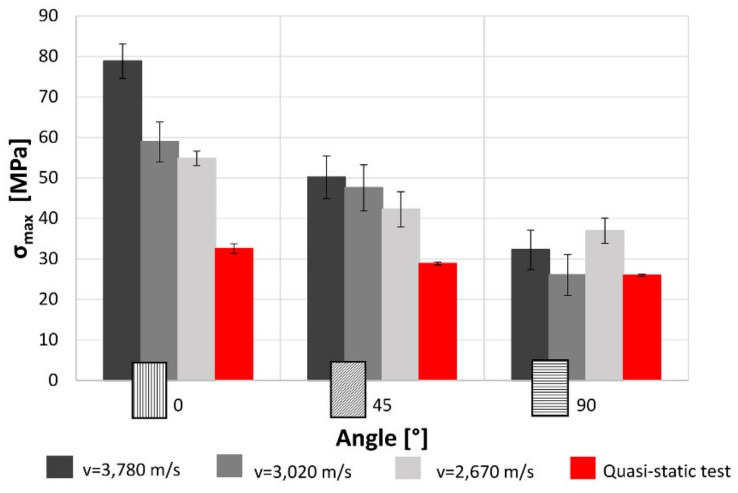
Effect on the maximum stress of the raster angles and impact speeds for multilayer specimens.

In Figure 15, it can be observed that increasing the raster angle there is a reduction of the maximum stress. This is true for each impact test speed. Moreover, for the 0° specimens, as already pointed out in Figure 11, there is a significant reduction of the maximum stress decreasing the impact speed. This reduction become less clear for the 45° specimens and it is statistically negligible in the 90° samples. The red bands are the results obtained from quasi-static tensile tests. The results from this are quite lower than the dynamic values. Generally, the multilayer specimens show an increase in maximum stress in comparison with single-layer specimens. This may be due to the partial overlap of the different layer and, consequently, the reduction in the internal voids that improve the mechanical characteristic of the material. Furthermore, according to the results obtained for single-layer specimens, the value of the maximum stress is function of the raster angle but it is not related with the impact velocity except that printed at 0° tested at maximum velocity. In the condition, indeed, the behaviour of the samples, due to the reduction of internal voids and to the layout of the material, is more similar to the row material behaviour [26,27].

**Figure 16:**
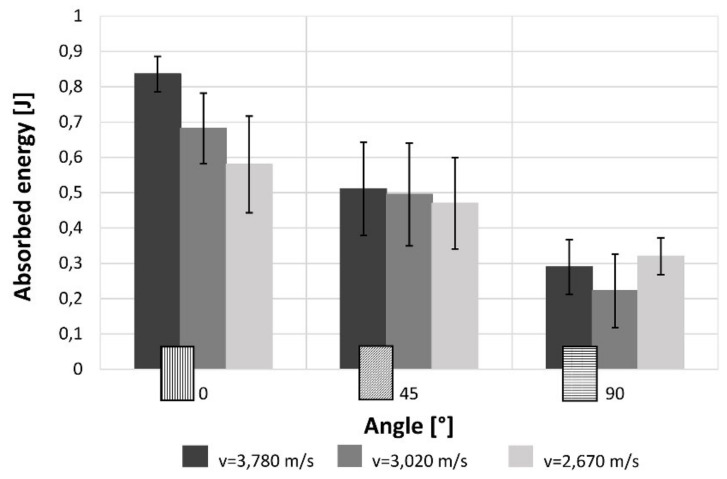
Effect on the absorbed energy of the raster angles and impact speeds for multilayer specimens.

Figure 16 highlights that the trend of the absorbed energy is comparable to the maximum stress trend. Indeed, increasing the raster angle there is a great reduction of the absorbed energy. Also in this case, the change due to the impact speeds are much more important in the 0°, become smaller in the 45° case and are negligible in 90° samples. Furthermore, the values of the energy absorbed during high strain rate tests is clearly lower in comparison with that measured under quasi-static condition because of the elasto-plastic behaviour of the material.

The stress–strain curves (Figure 5, Figure 6 and Figure 7 and Figure 12, Figure 13 and Figure 14) were obtained averaging the experimental data point by point imposing the same starting time, while the maximum of stresses is not always aligned. For these reasons, some minor differences in the comparison with data presented in Figure 7 and Figure 8 and Figure 14 and Figure 15 can be found. However, this graph is useful to understand the mechanical behaviour under high strain rate load of the parts obtained by FFF.

**Figure 17:**
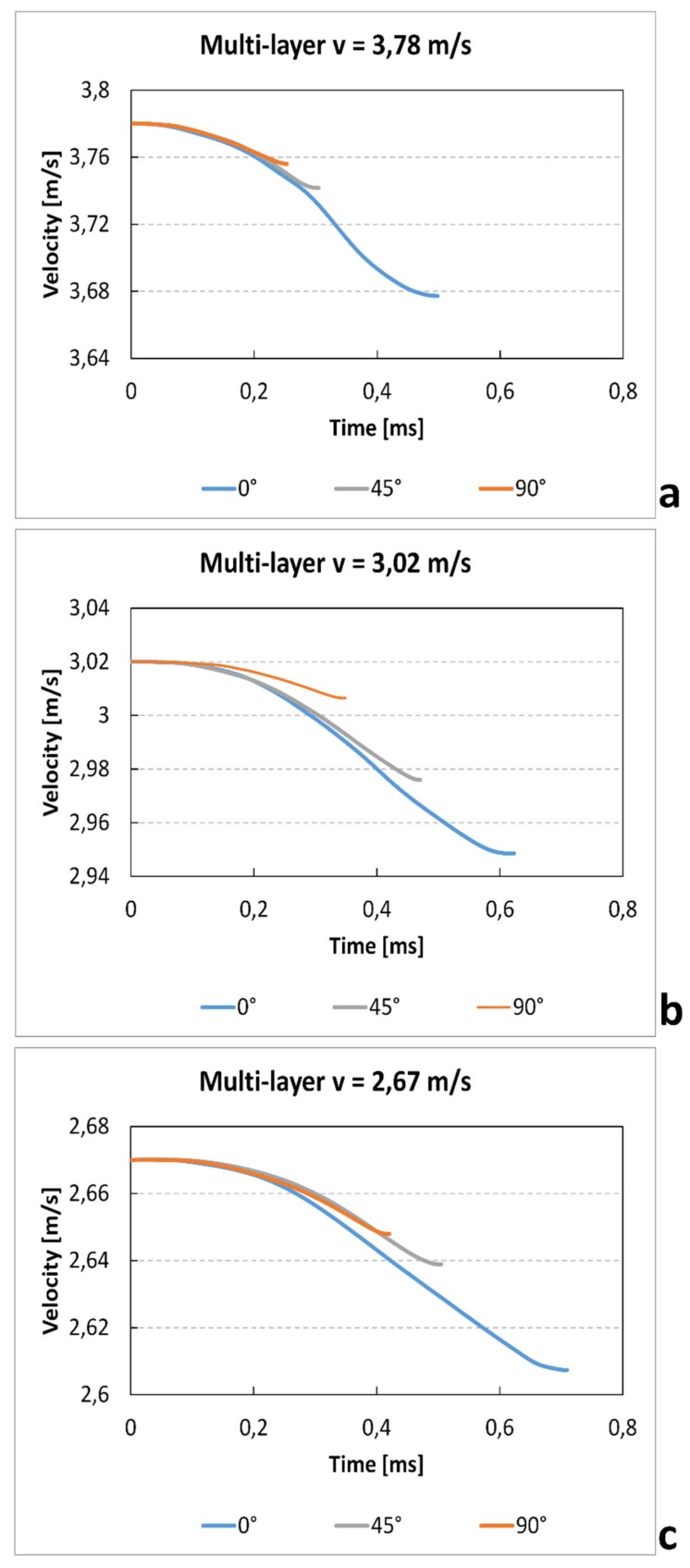
Multilayer velocity–time curves for different raster angles and velocities.

Similarly to the single-layer tests, the values of velocity–time for different raster angles and impact speeds are recorded in Figure 17. Also in this case, the results show a clear increase of time test and a reduction gradient velocity with increase of impact speed. Furthermore, likewise to the single-layer specimen, those printed with 0° raster angle show a higher duration of test. In fact, in this direction, the beads parallel to the load increase the strength of the samples

**Figure 18:**
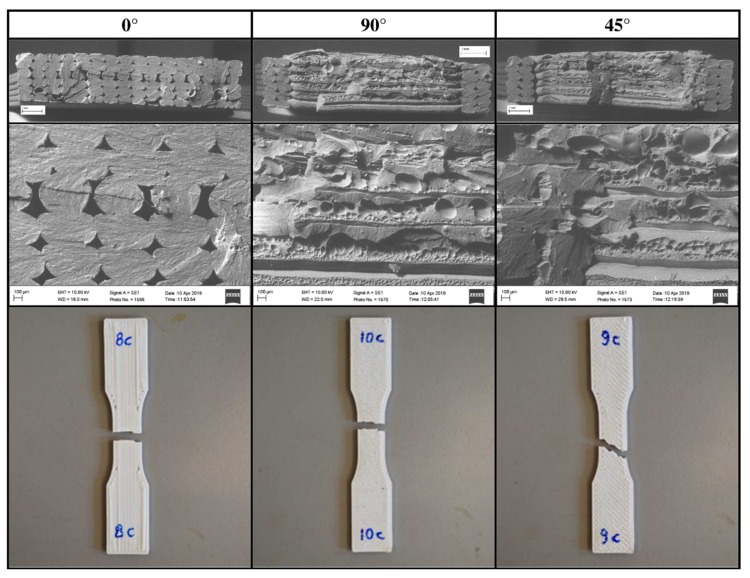
Fracture section for the multilayer specimens.

The fracture surfaces for the multilayer specimens (Figure 18) show that the fracture path is related to the raster orientation. But, while the 0° specimens show, as do the single-layer tests, tensile failure of individual fibres, the 45° and 90° specimens show a mixed-mode breaking surface. In fact, in both cases it easy to recognise both filament rupture and zones of breaking of the adhesion between two filaments. This could explain why, especially under static or at lower speeds loads, the difference in maximal stress between 0° and 90° is lower in multilayer specimens than in single-layer specimens.

Furthermore, microscopic images highlight the presence of voids between beads, with a reduction of the net section and of the real density of the component, but also the presence of cavity at the interface between beads. This second kind of defect, probably related to the extrusion temperature, can reduce the adhesion force between filaments.

## 4. Conclusions

The results for both the single-layer (SL) and multilayer (ML) specimens show that when increasing the raster angle from 0° to 90°, there is a reduction in the maximum stress (−38% SL; −50% ML) and the absorbed energy (−58% SL; −60% ML). This is true for each impact test speed. Moreover, single-layer specimens do not show relevant statistical variation in force and absorbed energy with impact velocity (Figure 7 and Figure 8). On the contrary, some variation can be highlighted in multilayer specimens, but only the 0° samples show an evident correlation between impact velocity and force and absorbed energy. This effect could be due to the fact that in the 0° orientation all the load is supported by the base material pointing out some of its viscoelastic property, while, in the other cases, the fracture surfaces coincide with the bonding area between cordons that are more fragile.

Furthermore, both for SL and ML specimens, those printed with 0° raster angle show a longer duration of test. In fact, in this direction, the beads parallel to the load increase the strength of the samples, highlighting the similarity with the row material behaviour [26,27]. For example, Xiao [26] has measured for ABS under a 4.0 m/s impact load a maximum stress of 78 MPa and a load rising time of 0.51 ms. Those values are comparable with that measured for 0° ML specimen, tested in the present work, with an impact velocity of 3.78 m/s (σ_max_ = 75 MPa; t = 0.50 ms). From this point of view, the behaviour samples obtained with FFF technique are similar with that obtained with more classic production methods.

In conclusion, the FFF parts are clearly influenced in an important manner by the raster orientation, confirming the orthotropic behaviour also under dynamic loads. On the contrary, the effect of variation of the impact speed on peak force and absorbed energy is limited. For these reasons, a good design of the FFF parts should take into account the direction of the loads to optimise the orientation of the layers. Generally, an orientation parallel to the load direction guarantees an increase in the mechanical properties, although further study will be necessary to understand the response under combined loads.

## Figures and Tables

**Table 1:** Dimensions, physical and thermal properties of acrylonitrile–butadiene–styrene (ABS).

Description	Typical Value
Diameter	2.85 mm
Density	1.03 g/cm^3^
Tensile strength [13]	32 MPa
Strain at break	9%
Tensile modulus [13]	1.8 GPa
Printing temperature	220–270°C
Melting temperature	245°C ± 10 °C

**Table 2:** Speed of the striker related to the total mass.

Sample Sets	Speed (m/s)	Total Mass Striker (kg)	Striker Dimension (m)	Crosshead Mass (g)
A	3.78	3.492	0.4	120
B	3.02	5.492	0.4	120
C	2.67	6.992	0.4	120

**Table 3:** FFF process parameters.

Parameter	Value
Air gap (mm)	0
Layer thickness (mm)	0.35
Bead width (mm)	0.70
Number of contour lines	2
Nozzle diameter (mm)	0.4
Bed temperature (°C)	90
Nozzle temperature (°C)	225

**Table 4:** Single- and multilayer mechanical properties at different raster angles.

Orientation	Single-Layer			Multilayer		
	Young’s Modulus (GPa)	UTS (MPa)	Absorbed Energy (J)	Young’s Modulus (GPa)	UTS (MPa)	Absorbed Energy (J)
0°	1.79 ± 0.58	26.11 ± 2.37	0.25 ± 0.06	1.96 ± 0.21	30.19 ± 1.09	2.58 ± 0.06
45°	1.38 ± 0.11	11.97 ± 3.12	0.09 ± 0.05	1.76 ± 0.12	27.70 ± 0.48	4.08 ± 0.67
90°	1.15 ± 0.09	6.71 ± 0.36	0.02 ± 0.01	1.64 ± 0.10	25.23 ± 0.47	2.30 ± 0.22
